# The Impact of Using Nesting Care on Heart Rate, Oxygen Saturation, and Pain Among Premature Neonates in Neonatal Intensive Care Units in Saudi Arabia: A Quasi-Experimental Study

**DOI:** 10.7759/cureus.61775

**Published:** 2024-06-06

**Authors:** Ahmad Ismail, Sahar M Salaghor, Salmah A Alshomrani, Hanan Almodallal

**Affiliations:** 1 Nursing, Fakeeh College for Medical Sciences, Jeddah, SAU

**Keywords:** saudi arabia, nicu, pain, heart rate, oxygen saturation, neonate, premature, nesting, supportive positioning aids

## Abstract

Background: Supportive positioning may mimic the intrauterine environment and enhance neonates' physiological and developmental outcomes. Limited research in Saudi Arabia examined the effect of supportive positioning aids (nesting) on infant outcomes in neonatal intensive care units (NICUs).

Objective: This study compared nesting care to non-nesting care in the short-term outcomes of premature neonates (heart rate, oxygen saturation, and pain) in Saudi NICUs.

Methods: A quasi-experimental design compared two groups of premature neonates from two NICUs regarding their heart rate, oxygen saturation, and pain level. Nesting was used in the first group, and not in the second group. Seventy premature neonates (35 per group) were recruited. An independent t-test was used to compare the two groups.

Results: Heart rate was significantly lower in the nesting group than the non-nesting group at baseline and after procedures (136bpm and 139bpm vs 144bpm and 148bpm, P ≤ 0.05). The pain level was significantly lower in the nesting group than the non-nesting group at baseline and after procedures (3.7 and 3.8 vs 4.7 and 4.6, P ≤ 0.05). There was no significant difference between the two groups in oxygen saturation.

Conclusion: Nesting care supported premature neonates in the NICU. It helped stabilize the heart rate and pain. NICUs in Saudi Arabia would benefit from educating NICU nurses and informing NICU managers and policymakers of nesting care.

## Introduction

According to the World Health Organization (WHO), 13.4 million neonates were born prematurely in 2020 (before 37 completed weeks of gestation) [1]. Preterm birth complications are the primary leading cause of death among children under five years of age, responsible for approximately 900,000 deaths in 2019 [2]. In neonatal intensive care units (NICUs) in Saudi Arabia, preterm birth and its complications account for 51.5% of death cases [3]. NICUs typically admit premature neonates with low birth weight (less than 1.5 Kg) who experience breathing difficulties, heart problems, infections, birth defects, or require surgical interventions [4,5]. Prematurity is associated with several complications including most of the body systems such as respiratory distress syndrome (RDS), transient tachypnea, bronchopulmonary dysplasia, pneumonia, apnea, bradycardia, infection and sepsis, jaundice, intraventricular hemorrhage, hypothermia, immature gastrointestinal system and necrotizing enterocolitis, anemia, patent ductus arteriosus, retinopathy of prematurity, musculoskeletal and neurodevelopmental [6-12].

Proper positioning of the premature neonate in the NICU is important to reduce the complications associated with prematurity, especially musculoskeletal and neurodevelopment complications [13-15]. The proper position of the premature neonate is the position that mimics the position inside the womb where the fetus is confined to an enclosed space with well-defined boundaries [16]. In the NICU, using the developmentally supportive positioning for premature neonates is essential for optimizing neuromotor and musculoskeletal development [15,16]. Proper positioning may also help maintain the stability of premature neonates' physiological signs, such as heart rate, oxygen saturation, and pain. However, there is a gap between theory and practice regarding developmental positioning, which is not a standardized intervention for premature neonates in all NICUs [13,16,17]. Nurses in the NICU usually place premature neonates in the supine and prone positions without considering the use of developmental positions [13].

Positioning devices and tools are one option for providing a proper position for premature neonates, such as “nesting care.” Nesting care is developmental care used in the NICU due to its simplicity, availability, and no restrictions to use. Nesting care results in many benefits [17]. Nesting care uses a rolled fabric around the neonate to totally contain the movement of the neonate from head to toe [18]. It adds boundaries around the neonate which reduces the sudden movement and excessive expansion of limbs. It also maintains the neonate in a curved limb position and improves the sleep quality of the newborn [13,19].

Many studies have been conducted to assess the effect of positioning aid devices on newborn outcomes, especially premature neonates, as these devices may mimic intrauterine life. The results of these studies were variable. Many benefits were observed when using positioning tools like nesting. It would improve sleep, oxygen saturation, pain level, comfort, and stress in premature neonates [17,18,20-23]. Research also identified facilitators and barriers to using positioning aids, including nesting care. The facilitators include healthcare professionals' knowledge and training, leadership and support, and adequate infrastructure. The barriers include healthcare professionals' lack of knowledge of developmentally supportive care, cultural norms, and the absence of policies or guidelines [24]. In Saudi Arabia, there is a lack of research on the use of supportive positioning tools and their impact on premature neonatal outcomes in the NICU. Therefore, this study aimed to compare nesting care to non-nesting care in the short-term outcomes of premature neonates (heart rate, oxygen saturation, and pain) in Saudi NICUs.

## Materials and methods

Research design

This research study employed a quasi-experimental design to compare the physiological outcomes (heart rate, oxygen saturation, and pain level) of two groups of premature neonates from two NICUs in Saudi Arabia. One group received nesting care, while the other did not. The data were collected between January and April 2023.

Setting

This study was conducted in two NICUs from two hospitals in Jeddah, Saudi Arabia (one government hospital and one private hospital). In the government hospital (King Abdullah Medical Complex, Jeddah), nesting care was applied as routine care for all premature neonates. In the private hospital (Dr. Soliman Fakeeh Hospital, Jeddah), nesting care was not applied. This allowed us to compare the two groups of premature neonates. Characteristics of the neonates were collected to assess the differences between the two groups in terms of these characteristics to reduce the effect on the outcomes of this study (heart rate, oxygen saturation, and pain).

Study participants and sample

The sample of this study consists of premature neonates in Saudi Arabia. A convenience sample of premature neonates was recruited for this study. The neonates were selected based on the intervention received (nesting care vs traditional). We selected one hospital (government) where nesting care was applied to all premature neonates and a private hospital where nesting care was not applied. The researchers did not assign the intervention to the interventional group, as the nesting care was applied as routine care. The sample was convenient, and so was the selection of the participants. As this study compared two independent groups (nesting vs traditional care), the sample size calculation depends on the calculation of the effect size of the independent t-test (Cohen d). A pilot study of 20 participants (10 per group) was conducted to determine the effect size. The effect size was calculated based on one parameter (heart rate). The effect size was 0.61. Using a sample size calculation software (G*Power3), the required sample size was 35 neonates per group at a power level of 80% and a P of 0.05. This study recruited two equal groups of premature neonates based on the effect size calculation to ensure that the sample size adequately represented the population. In addition, an equal group sample allows for reaching the power and reduces the chance of bias.

Data collection procedure

Ethical approvals were obtained from the respective institutions. Data collection tools were developed, including a demographic section and a section on patients' physiological outcomes before, during, and after 10 minutes of the medical intervention. Consent forms were obtained and signed by parents. Data were collected from two NICUs, one public and one private in Jeddah, Saudi Arabia. The primary investigator visited the sites and was granted permission to begin collecting data from the unit after presenting the study's purpose and benefits to the unit managers. All patients admitted to the NICU who were less than 37 weeks of gestation were included in the study. Data were collected every week, and information was recorded on a spreadsheet.

Data collection tool

The data collection sheet was developed to meet the study goal. It included two main sections (demographic and physiological items of the neonates). Demographic items of the neonates included weight in grams, admission age in weeks, hospitalization days, gender, Kangaroo Mother Care (KMC) use, reason for admission, intervention, medication use, respiratory status, and feeding type. Physiological items included heart rate, oxygen saturation, and pain level. Heart rate and oxygen saturation were recorded using the cardiac monitor in the unit (Philips IntelliVue MX800- Bedside patient monitor; Philips, Amsterdam), and the pain level was assessed using the premature infant pain profile scale (PIPP). The PIPP is a seven-item multidimensional scale used to assess acute pain in neonates, especially premature ones. The PIPP includes three behavioral items, two physiological, and two contextual. Items are scored on a four-point scale (0, 1, 2, and 3). A higher score indicates a higher pain level [25]. 

Data analysis

The data was analyzed using IBM SPSS Statistics for Windows, Version 26 (Released 2019; IBM Corp., Armonk, New York, United States). Data screening was done to ensure the integrity of the data. Data were analyzed for frequency, percentages, and means. The independent t-test was used to compare the two groups' heart rate, oxygen saturation, and pain level. The independent t-test, chi-square, and Fisher exact tests were used to compare the demographics of the two groups.

Ethical considerations

Ethical approval from the Research Ethics Committee of Fakeeh College for Medical Sciences (256/IRB/2022) and the Directorate of Health Affairs in Jeddah (A01523) was obtained. Consent forms were obtained from the neonates' parents, explaining the study's purpose, benefits, and harms. Participation was voluntary. Parents had the right to withdraw their neonates from the study at any time. Data were coded, and personal information was kept confidential and protected by a password. Nobody would have access to data but the researchers. Parents were assured that their neonates’ data would be presented and published in aggregates, and data would be destroyed by a secure deletion process after five years of the results’ publication.

## Results

This study recruited 70 premature neonates, with 35 neonates receiving nesting care at a government hospital and 35 receiving traditional care at a private hospital. The traditional care group's mean weight was 1655 grams, gestational age 31, and six days of hospitalization. The majority were admitted for prematurity or/and RDS (91%) and supported by respiratory supporting devices (63%). Near half were males (57%) and received their feeding via an oro-gastric tube (51%). The most common procedures they received were feeding (34%), venous access (14%), suction (11%), and positioning (11%). Twenty-three percent received sedatives and opioids. For the nesting care group, the mean weight was 1574 grams, the mean gestational age was 30 weeks, and seven days of hospitalization. The majority were admitted for prematurity or/and RDS (97%) and received feeding orally or via an oro-gastric tube (69%). Near half were females (54%) and on room air (51%). The most common procedures they received were feeding (31%), feeding and nappy care (28%), and positioning (14%). Fourteen percent received opioids and sedation. The two groups had no significant differences in all variables (P ˃ 0.05) (Table 1).

**Table 1:** Demographic Characteristics of the Premature Neonates t: Independent t-test; X^2^: Chi-Square; IUGR: Intrauterine Growth Retardation; RDS: Respiratory Distress Syndrome; HFNC: High-Frequency Nasal Cannula; CPAP: Continuous Positive Airway Pressure; ETMV: Endotracheal Tube with Mechanical Ventilator; NPO: Nothing per Mouth; KMC: Kangaroo Mother Care; F: Frequency; NA: Not Applicable

Item	Traditional Group Ranage (Mean)	Nesting Care Group Range (Mean)		Test	P
Weight in grams	880 to 2800 (1655)	1000 to 2610 (1574)		t	0.47
Admission age in weeks	25 to 34 (31)	23 to 35 (30)		t	0.45
Hospitalization days	1 to 19 (5.5)	1 to 15 (7.1)		t	0.11
Item	Traditional Group F (%)	Nesting Care Group F (%)		Test	P
Gender					
Male	20 (57.14%)	16 (45.71%)		X^2^	0.34
Female	15 (42.86%)	19 (54.29%)			
Reason for Admission					
Prematurity	12 (34.29%)	21 (60.00%)		Fisher Exact	0.09
Prematurity and RDS	20 (57.14%)	13 (37.14%)			
Prematurity and IUGR	3 (8.57%)	1 (2.86%)			
Intervention					
Nappy care	3 (8.57%)	4	11.43%	Fisher Exact	0.15
Feeding	12 (34.29%)	11	31.43%		
Nappy care and feeding	3 (8.57%)	10	28.57%		
Suction	4 (11.43%)	0 (0.00%)			
Venous Access	5 (14.29%)	5 (14.29%)			
Skin prick	2 (5.71%)	0 (0.00%)			
Positioning	4 (11.43%)	5 (14.29%)			
Bathing	1 (2.86%)	0 (0.00%)			
Chest tube insertion	1 (2.86%)	0 (0.00%)			
Medication					
None	8 (22.86%)	9 (25.71%)		Fisher Exact	0.37
Sedatives	5 (14.29%)	5 (14.29%)			
Opioids	3 (8.57%)	0 (0.00%)			
Others	19 (54.29%)	21 (60.00%)			
Respiratory status					
Room air	13 (37.14%)	18 (51.43%)		X^2^	0.8
HFNC	8 (22.86%)	4 (11.43%)			
CPAP	3 (8.57%)	8 (22.86%)			
ETMV	11 (31.43%)	5 (14.29%)			
Feeding Status					
NPO	13 (37.14%)	11 (31.43%)		X^2^	0.12
Oral	4 (11.43%)	11 (31.43%)			
Orogastric Tube	18 (51.43%)	13 (37.14%)			
KMC					
Yes	0 (0.0%)	0 (0.0%)		NA	NA
No	35 (100%)	35 (100%)			

There was a statistically significant difference in heart rate at baseline and after procedures between the nesting and traditional care groups (136 and 139 vs 144 and 148 beats per minute, respectively) (P ˂ 0.05). There was also a statistically significant difference in the pain score at baseline and after procedures between the nesting care group and the traditional care group (3.7 and 3.8 vs 4.7 and 4.6, respectively) (P ˂ 0.05). There was no statistically significant difference between the two groups in oxygen saturation (P ≥ 0.05) (Table 2).

**Table 2:** Heart Rate, Oxygen Saturation, and Pain of Neonates in Traditional and Nesting Care Groups Bold: Significant value, P < 0.05

Parameter	At Baseline		During the Intervention		10 Minutes after the Intervention	
Heart Rate Beats Per Minute	Range (Mean)	P	Range (Mean)	P	Range (Mean)	P
Traditional group	110 to 188 (144)	0.024	78 to 194 (153)	0.14	110 to 175 (148)	0.018
Nesting group	115 to 166 (136)		124 to 181 (159)		115 to 184 (139)	
Oxygen Saturation						
Traditional group	91 to 100 (97)	0.77	72 to 100 (95)	0.66	90 to 100 (96)	0.73
Nesting group	90 to 100 (98)		80 to 100 (94)		91 to 100 (97)	
Pain						
Traditional group	2 to 7 (4.7)	0.001	1 to 14 (5.5)	0.31	3 to 8 (4.6)	0.017
Nesting group	2 to 5 (3.7)		2 to 15 (4.7)		1 to 6 (3.8)	

## Discussion

Two equal groups of premature neonates were recruited for this study (nesting care vs traditional). The two groups were similar in all demographic characteristics. This limits the effect of their effects on the outcomes of this study (heart rate, oxygen saturation, and pain). This study provides empirical evidence of the impact of nesting care on some physiological outcomes. This study shows that neonates who received nesting care achieved lower heart rates and pain than those who did not. 

Several research studies have assessed the impact of nesting care as a standalone intervention on neonatal outcomes. A randomized controlled trial found that nesting care produced a superior effect on sleep duration than swaddling among premature neonates in the NICU in India [23]. In the same research, there was no significant difference between premature neonates in nests and swaddled physiological parameters, including heart rate, respiratory rate, oxygen saturation, and temperature. Another quasi-experimental research found that nesting care resulted in many positive physiological, behavioral, and neurological outcomes among premature neonates in the NICU in Egypt. Based on the previous study, premature neonates who received nesting care achieved more stable temperature and oxygen saturation, less crying, and more sleeping time than those who did not [26]. Consistent with our study, an experimental study from Turkey found that premature neonates who received nesting care achieved higher oxygen saturation and lower pain levels than those who did not [27]. Our study adds to the previous three studies that nesting care would positively affect the heart rate of premature neonates.

Several research studies assessed the impact of nesting care with other interventions on neonatal outcomes, such as positioning. One experimental study indicated that nesting in a prone position promoted comfort and reduced stress and pain among premature babies in the NICU during heel lancing in Turkey. This study also found that salivary cortisol levels were significantly lower in the nesting in prone position neonates than in the supine position [21], indicating lower stress levels among neonates [21,28]. A crossover design study found that nesting with fixation in a prone position resulted in a longer sleep period and more stability in oxygen saturation among low-birth-weight infants in Jakarta [20]. In our study, the nesting group babies were recruited regardless of their position (prone or supine). Our study showed that nesting care positively affected heart rate and pain outcomes regardless of the neonatal position. Given the evidence of using combined methods, further studies are necessary in Saudi Arabia to examine the differences in using these methods separately and combined.

Some research found that other interventions have a greater effect than nesting care on some parameters, such as pain and stress. One study found that KMC with dextrose 50% resulted in more pain relief than nesting care with a supine position among late premature neonates who have undergone heel pricks [29]. Another study found that hammock position resulted in lower stress levels among premature neonates than nesting care after nappy care [18]. Nurses in the NICUs in Saudi Arabia may consider using nesting care with other interventions, considering the patient's condition. An interventional study conducted to improve NICU staff use of nesting care with positioning found that educational intervention was highly effective in improving both the instrumental and cognitive use of nesting care and positioning. After the intervention, the NICU nurses’ use of proper developmental positions for premature neonates increased from 63% to 91%. Cognition increased from 58% to 92% [30].

Although this research provides important information about the impact of nesting care on neonatal outcomes in Saudi Arabia, it has four limitations. First, this study recruited a convenience sample of neonates from only two NICUs in Saudi Arabia (one governmental and one private), which may affect the generalizability of the results beyond these two settings, given the differences related to the setting. Second, this study did not assess the competency of healthcare professionals (such as NICU nurses) in applying nesting care, so describing how nesting care is applied would be helpful. Third, due to sample size, this study used bivariate analysis to compare the nesting care and control group, so the findings should be interpreted with caution as confounding variables (e.g., setting type) were not controlled in the analysis. Fourth, there was no randomization in assigning the neonates to nesting and non-nesting groups. Therefore, future research may recruit more neonates from various NICUs in Saudi Arabia, considering using random sampling with random assignment of the participants. Future research also needs to assess the competency and education of NICU healthcare professionals in applying positioning aids in the NICU.

## Conclusions

Nesting care in the NICU has been shown to support neonates, resulting in lower heart rate and pain among premature neonates. NICUs in Saudi Arabia would benefit from educating NICU nurses and informing NICU managers and policymakers of nesting care. Future research should recruit more neonates from various NICUs in Saudi Arabia, considering using random sampling with random assignment of the participants.
